# Environmentally Sustainable Cement Composites Based on End-of-Life Tyre Rubber and Recycled Waste Porous Glass

**DOI:** 10.3390/ma12203289

**Published:** 2019-10-10

**Authors:** Andrea Petrella, Rosa Di Mundo, Sabino De Gisi, Francesco Todaro, Claudia Labianca, Michele Notarnicola

**Affiliations:** Dipartimento di Ingegneria Civile, Ambientale, Edile, del Territorio e di Chimica, Politecnico di Bari, Via E. Orabona 4, 70125 Bari, Italy; rosa.dimundo@poliba.it (R.D.M.); sabino.degisi@poliba.it (S.D.G.); francesco.todaro@poliba.it (F.T.); claudia.labianca@poliba.it (C.L.); michele.notarnicola@poliba.it (M.N.)

**Keywords:** cement composites, recycled waste porous glass, end-of-life tyre rubber, safe production, thermal insulation, mechanical resistance

## Abstract

In this paper, environmentally sustainable cement mortars were prepared with end-of-life tyre rubber (TR) and recycled waste porous glass (PG) as aggregates in order to obtain lightweight products characterized by renewable and not-pretreated materials specifically for indoor applications. The secondary raw materials were added as partial and/or total replacement of the conventional sand aggregate. The resulting lightweight specimens were characterized by rheological, mechanical, thermal, microstructural and wettability tests. Fine tyre rubber aggregates affected the cohesiveness of the composites, as opposite to coarse tyre rubber and porous glass. The flexural and the compressive strengths of the porous glass samples were higher than the tyre rubber samples because of the higher stiffness and good adhesion of the glass to the cement paste as observed by microstructural observations. On the contrary, an unfavorable adhesion of the tyre aggregates to the cement paste was observed, together with discrete cracks after failure without separation of the two parts of the specimens. The latter result can explain the best results obtained by tyre rubber mortars in the case of impact compression tests where the super-elastic properties of the elastomeric material were evidenced by a deep groove before complete failure. Moreover, the thermal conductivity decrease of the lightweight porous TR and PG composites was in the range of ~80–90% with respect to the sand-based samples, which suggests that they can be used as plasters and masonries, and, in the case of tyre rubber specimens, outside applications are not excluded as observed from the wettability tests.

## 1. Introduction

The recycling of industrial by-products is an ever rising issue in the sustainable waste management field. Indeed, many studies have focused on the conversion, after appropriate procedures, of these secondary raw materials into a new resource that would otherwise be landfilled. In this respect, over the last years, industrial waste recycling and reuse have become important environmental challenges that many countries are facing in order to reduce overall costs and negative environmental impacts [1,2,3,4,5,6,7,8,9,10]. 

Waste glass and tyre rubber are among the most recycled secondary raw materials from industrial and municipal activities, accordingly, various investigations, mainly in sustainable construction technology, have been carried out with the aim to face the problems relative to the disposal space limitations of these by-products associated with the increasing costs [8,11,12,13,14,15,16,17].

In recent years, the amount of waste glass has gradually increased due to the widespread development of urban areas and industries. After the forming process, different types of glass products can be obtained, specifically container glass, flat glass, light bulbs, fluorescent and cathode ray tubes. The biopersistence and chemical inertia of these products lead to long-term accumulation. Accordingly, in the ambit of environmentally sustainable management policies, glass needs to be reused and/or recycled. This material can be indefinitely recycled by preserving the original properties [18,19], and accordingly, the products collected during the sorting operations can be used for the production of abrasives, reflective paints for highways, cullets in glass production, lubricants, additives, fractionators, in road beds and fiberglass production [19,20,21,22,23]. 

In the last 20 years, recycled glass has been widely used in construction materials, specifically in bricks, normal concrete, pavement materials and asphalt concrete [19,24,25,26,27,28], which is a practice that reduces landfill operations and consumption of natural resources, and also minimizes greenhouse emissions [18,29]. In this respect, it is worth saying that in the case of building materials, recycled glass as aggregate in structural concrete is still not widely used.

The growing amount and disposal of waste tyre rubber has become an environmental issue in many countries. Every year, millions of end-of-life tyres are discarded all over the world and stockpiled tyres represent a threat to human health and the environment through air, water and soil pollution, with its associated economic and social risks [15,30]. The volume of waste, which is globally produced, makes management of the accumulated rubber very hard, with potential fire risks. Tyre burning, although easy and cheap, represents an extremely dangerous method of disposal because fires are difficult to be extinguished and uncontrolled emission of hazardous compounds and potentially toxic gases are released in air. This is very dangerous to humans, animals and plants, and causes ground and surface water contamination generated by the oils and residue ashes left after burning [31,32]. Tyre rubber may be also used as fuel after carbon black production but this solution is not economically advantageous because this material has lower quality and higher costs as compared to conventional fuels [15].

Due to the biopersistence and chemical inertia of waste rubber, recycling operations are rising issues in the sustainable waste management field, as an alternative to landfilling, along with the awareness that new products can be produced with different properties with respect to the original materials.

Tyre rubber can be used for applications in civil and non-civil engineering, for example in erosion control, earthquake shock-wave absorption, road construction as a modifier in asphalt paving mixtures, in breakwaters, in crash and sound barriers, in reefs, playground equipment, as a fuel in cement kilns or for electricity production after incineration [14,16,33,34,35]. 

Over the last few years, waste tyre rubber incorporation into cement concrete has been considered one of the most effective, cheap and eco-friendly recycling solutions because it contributes to reducing the cost of some natural aggregates, the great volume of tyre waste, and the emission of toxic compounds and carbon dioxide by preventing tyre fires [36,37,38,39].

The main purpose of the present research was to prepare and characterize, by physico-mechanical procedures, eco-friendly non-structural cement composites based on inorganic and organic by-products of recycled waste porous glass (PG) and end-of-life tyre rubber (TR). The secondary raw materials were added as partial and/or total replacement of the conventional sand aggregate, which was made on a volume basis rather than on a weight basis due to the low specific weight of both waste materials. The specimens were characterized by rheological, mechanical, thermal, porosimetric, microstructural and wettability tests. The aim was to obtain lightweight thermo-insulating composites specifically for indoor applications in perfect agreement with the current policies of environmental sustainability. They are also cost-effective because they are prepared through a cheap process where the renewable aggregates are not pre-treated (no addition of chemicals to improve adhesion to the cement paste) and the mixture preparation does not require complex manufacturing processes or expensive procedures.

## 2. Experimental Part

### 2.1. Materials and Mortar Specimens Preparation 

CEM II A-LL 42.5 R (limestone Portland cement, R_c (2 days)_ > 25.0 MPa, R_c (28 days)_ > 47.0 MPa, 3100–4400 cm^2^/g Blaine specific surface area) was provided by Buzzi Unicem (Barletta, Italy) and used for the preparation of the cement mortars [40]. Conventional sand (normalized) was characterized as clean, isometric and rounded in shape grains in the 0.08–2 mm size range (1660 kg/m^3^) and provided by Societè Nouvelle du Littoral, Leucate, France [41,42]. End-of-life tyre rubber (TR) (0–0.5 mm and 0.5–2 mm size range, 460 kg/m^3^ and 500 kg/m^3^, respectively) and recycled porous waste glass (PG) (0.5–2 mm size range, 300 kg/m^3^) were provided by Maltek Industrie S.r.l., Terlizzi, Bari, Italy. PG is a sodium calcium silicate glass (71% SiO_2_, 9% CaO, 14% Na_2_O, 3% Al_2_O_3_, 2% MgO, 1% K_2_O) obtained from separate collection and separation of municipal and industrial solid wastes. Preliminary cleaning and crushing of the raw materials is followed by the addition of a porosizing agent at temperatures of 900–1300 °C, which induces a controlled porosity of the resulting beads, thus showing a specific weight in the 200–900 kg/m^3^ range. TR and PG were added as partial and/or total replacement of the conventional aggregate, which was made on a volume basis rather than on a weight basis due to the low specific weight of both waste materials. In the present case, the total volume of aggregate was set at 500 cm^3^ in order to preserve an acceptable workability of the mixture. For this purpose, another sand reference (sand, sample 2) with the same aggregate volume (500 cm^3^) and with a 0.5–2 mm sand size range (1880 kg/m^3^) was prepared. Table 1 and Table 2 report the aggregate types used for the mortar preparation and the composition of the conglomerates. In the present case, the composites were prepared with a water/cement ratio equal to 0.5, specifically with 225 g of water and 450 g of cement; dosages that were chosen according to the standard protocol [41] for the normalized mortar preparation, showing a plastic behavior. After the mixture, the rheology of the fresh mixtures was evaluated by the flow-test [43]. The mortars were placed inside a truncated cone shape ring. After demolding, fifteen hits in fifteen seconds were applied. Flow data were calculated through the following empirical equation after evaluation of the diameters of the mixture before (D_i_) and after (D_m_) the test:

(1) $$\%flow=\frac{\left[\left(D_{m}-D_{i}\right)\right]}{D_{i}}*100$$

The percentage increase of the diameter of the non-consolidated sample over the base diameter represents the flow of a specimen.

Successively, all the specimens were molded in the form of prisms (40 mm × 40 mm × 160 mm) for the flexural and compressive tests and cured in water for 7, 28, 60 and 90 days after demolding [41]. Moreover, the specimens were molded in the form of cylinders for thermal (φ = 100 mm; H = 50 mm) and impact resistance (φ = 150 mm; H = 60 mm) tests and cured in water for 28 days after demolding. 

Porosimetric measurements of the resulting mortars were carried-out by Ultrapyc 1200e Automatic Gas Pycnometer, Quantachrome Instruments, Boynton Beach, FL, USA. In this respect, helium gas penetrates the finest pores of the material and the results were the average of three measurements performed on three specimens of the same type (see Table 2). 

### 2.2. Microscopical Characterization

A scanning electron microscope (SEM) was used to show magnified images of the aggregates and of the cement composites. For this purpose, a FESEM-EDX Carl Zeiss Sigma 300 VP (Carl Zeiss Microscopy GmbH, Jena, Germany) electron microscope was used and the samples were sputtered with gold after immobilization onto aluminum stubs (Sputter Quorum Q150 Quorum Technologies Ltd, East Sussex, UK). The elemental composition of the different organic and inorganic areas of the samples was obtained by energy dispersive X-ray (EDX) analysis (Oxford Instruments, X-Max 20, Abingdon-on-Thames, UK). Specifically, sand composition was: C (4%), O (52%), Si (35%), Ca (2%), end-of-life tyre rubber composition was: C (25%), O (70%), S (1.5%), recycled porous waste glass was: Na (14.8%), Mg (2.1%), Al (3.5%), Si (66%), K (1%), Ca (12%), cement paste composition was: C (4.2%), O (40%), Si (7.6%), Ca (44%), Fe (1.5%), Al (2.5%). A homemade system (premier series dyno-lyte portable microscope and background cold lighting) allowed us to evaluate the wettability of the specimens, which was carried out after deposition of a drop of water onto the side and fracture surface of each sample.

### 2.3. Mechanical and Thermal Tests

The flexural and compression tests were carried-out by the use of a MATEST device, Milan, Italy. Compression strengths were obtained on twelve semi-prisms (loading rate in the range of 2400 ± 200 N/s), deriving from the flexural tests carried-out on six prisms (40 mm × 40 mm × 160 mm) (loading rate in the range of 50 ± 10 N/s) [41].

An impact resistance device was made according to the ACI Committee 544 [44]. Specifically, the test was carried-out by dropping a weight of 4.50 kg from a height of 45 cm above a steel ball (63 mm diameter) placed centrally on the upper surface of the specimen. The energy absorbed by the sample before the fracture was obtained after evaluation of the number of blows on the sphere. 

Thermal conductivity (λ) and thermal diffusivity (α) measurements were carried-out through an ISOMET 2104 device, from Applied Precision Ltd. (Bratislava, Slovakia). Before the tests, the mortars were dried to a constant weight, followed by final stabilization at room temperature. Specifically, a constant thermal flow was produced by a heating probe that was applied on the sample surface and the temperature was recorded over time. The thermal diffusivity and thermal conductivity parameters were obtained by comparison between the experimental temperature values and the analytical solution of the heat conduction equation [45].

## 3. Results and Discussion

### 3.1. Characterization of the Aggregates and Rheological Tests of the Mortars

**Figure 1:**
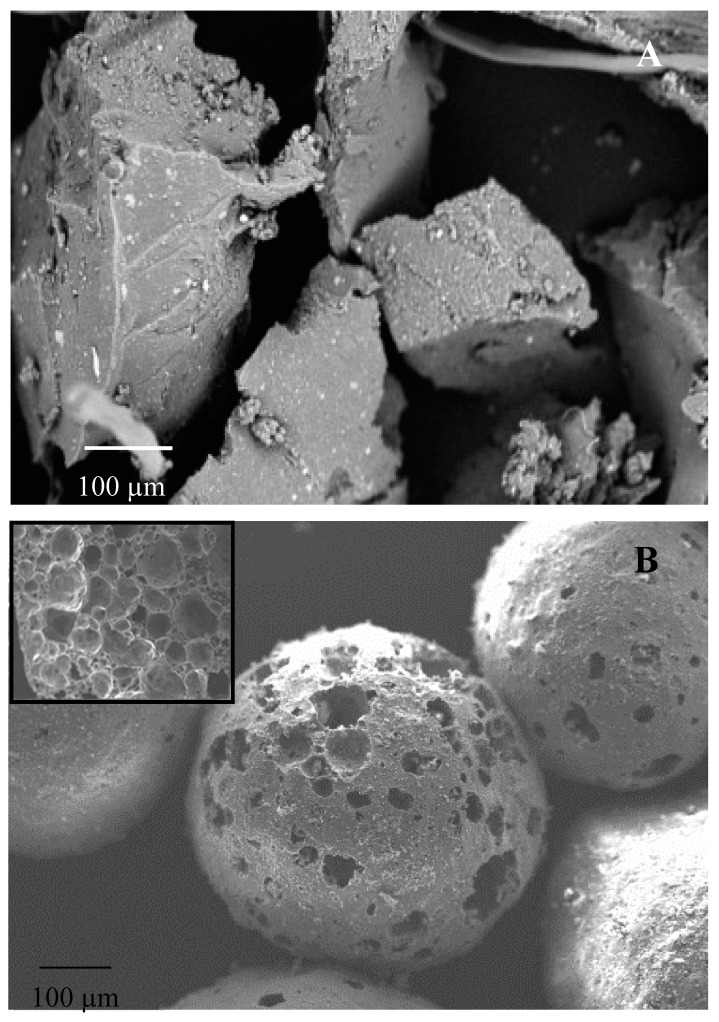
(**A**) Tyre rubber (TR) grain and (**B**) porous glass (PG) bead with evidenced porosity (in the inset: inner porosity).

Figure 1 shows the scanning electron micrographs (SEM) of tyre rubber grains and of PG beads. An intrinsic micro-scale texture of the elastomeric aggregates can be observed (Figure 1A), while the glass aggregates (Figure 1B) show a large open porosity together with a large closed porosity (inset Figure 1B). The properties of these secondary raw materials can explain the properties of the resulting mortars. In this respect, Table 2 shows that the TR and PG samples are lighter and with a much higher porosity than the references, while, among the lightweight composites, PG mortars were the most porous. 

**Figure 2:**
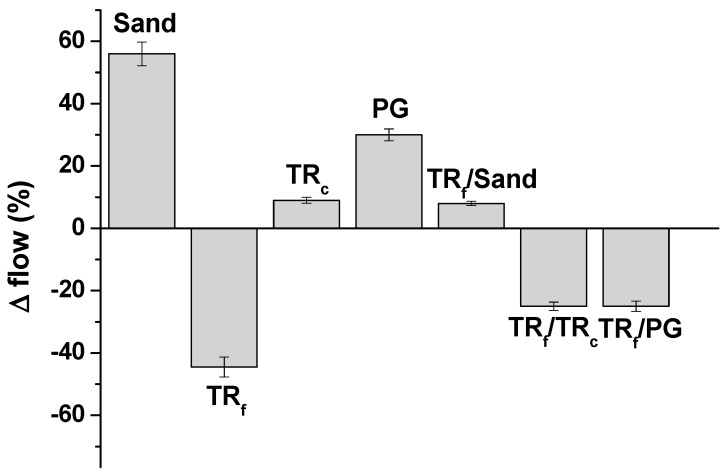
Flow-test results with respect to the normalized mortar (norm sample).

Flow-test measurements were carried-out on the mixtures in order to determine the consistency of the fresh specimens (Figure 2). 

Sample 2 (sand sample) showed a higher flow (+56%) than the control (normalized mortar (norm), with a plastic behavior) due to the lack of fines in the sand aggregate. The sample with bare fine tyre rubber aggregates (sample 3) showed a flow decrease in the range of 44.5% with respect to the reference [46], as opposite to sample 4 with bare coarse tyre rubber aggregates, which showed approximately the same flow as the control (+9%). The former result is ascribed to the absence of the fine aggregates, with a higher specific surface, which contributes to the decrease of plasticity and increase of cohesiveness of the specimen. Sample 6, with fine TR and coarse sand, showed a plastic behavior as the flow was similar to the control (+8%), while the PG composite (sample 5) showed a flow increase in the range of 30% because of the absence of fine aggregates. Finally, the presence of the finer TR fraction is associated with the decrease of workability of the samples 7 and 8 (−25%).

### 3.2. Mechanical Tests and Microscopical Characterization of the Mortars

**Figure 3:**
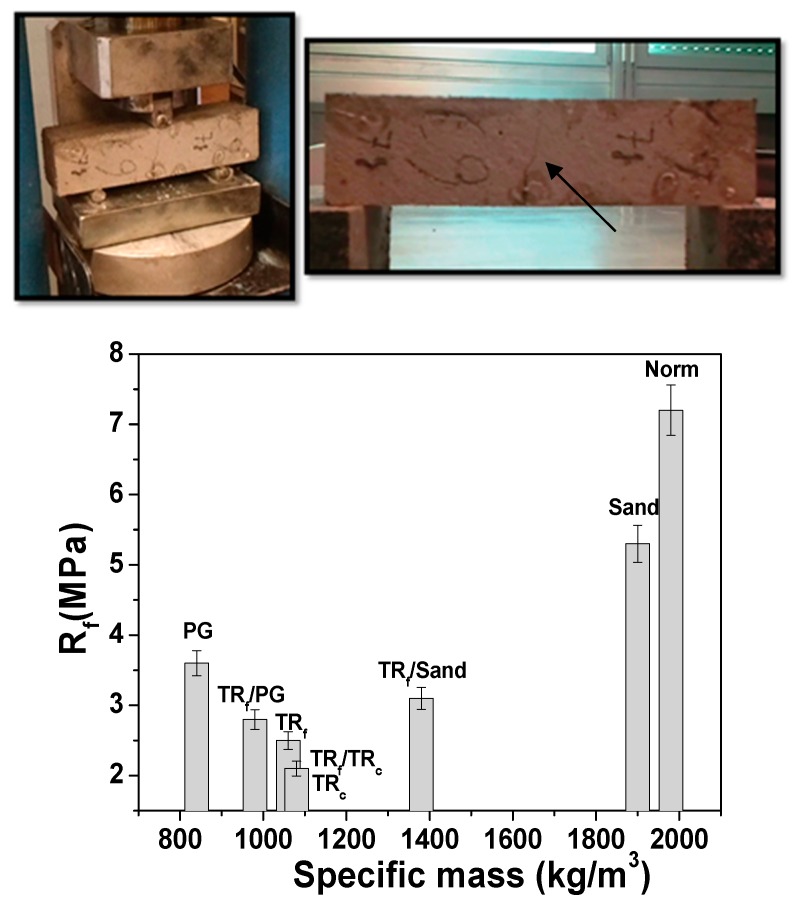
Flexural strengths of the cement mortars. On the top: discrete cracks after rupture in the TR specimens (evidenced by the arrow), with the two parts of the sample still connected by the tyre rubber.

**Figure 4:**
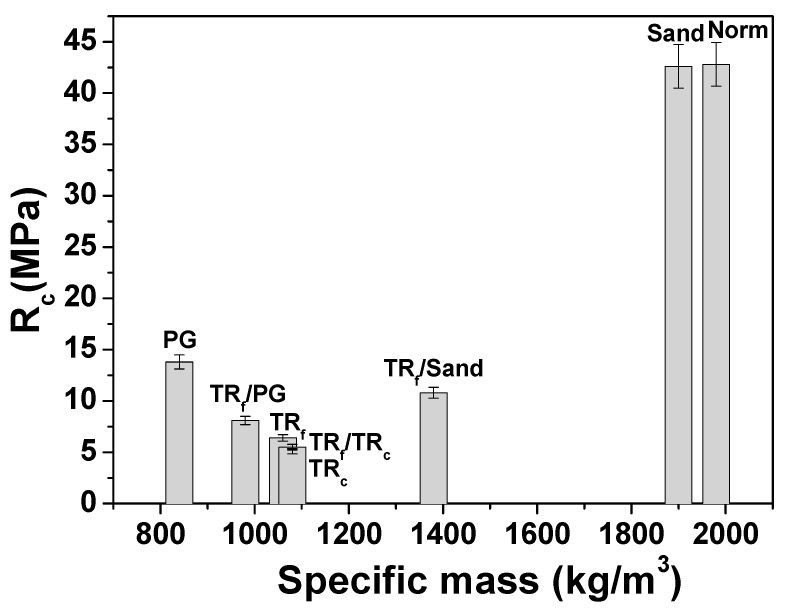
Compressive strengths of the cement mortars.

Figure 3 and Figure 4 report the flexural and compressive strengths of the samples. Reference sand-based mortars (samples 1 and 2) showed the best mechanical performances. In fact, the flexural and compressive strengths of all the unconventional and lightweight mortars were lower than the references, based on the more resistant sand aggregate.

As for specimens 3, 4 and 7, characterized by bare tyre rubber, the addition of the elastomeric waste disrupted the mineral skeleton of the mortars after the formation of voids in the composite, which sensibly reduced the specific weight of the samples. 

**Figure 5:**
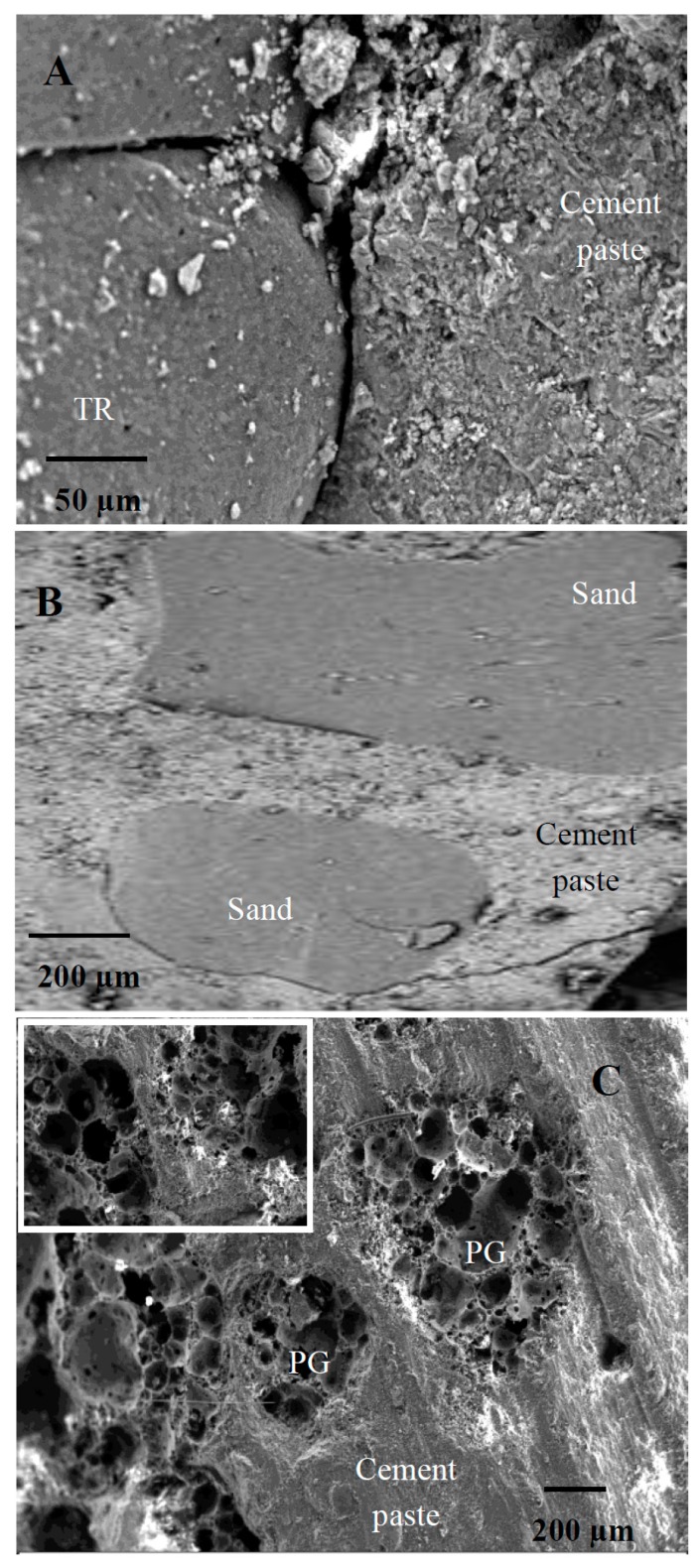
SEM images of: (**A**) cement/TR interface, (**B**) cement/sand interface, (**C**) cement/PG interface.

This effect depends on the organic/inorganic interface properties. In fact, an unfavorable adhesion of the aggregate to the cement paste [46,47,48,49,50,51,52] was observed and ascribed to the hydrophobic nature of the tyres rubber and to the completely different chemistry of the compounds present in the polymeric structure and in the inorganic matrix (Figure 5A). This evidence was not observed in the sand-based references where a good aggregate/cement paste adhesion was present (Figure 5B).

Accordingly, after total replacement of the sand volume, a decrease in the mechanical performances of these composites was ascribed to the low density of the TR grains and to the voids (entrapped air) created by the aggregate at the cement/TR interface during mixing, which can explain the decrease of the specific weight and an almost double porosity of these samples with respect to the references [46,47,48,49,50,51,52] (Table 2). 

Specifically, the flexural resistances of samples 3, 4 and 7 were 60–70% lower than the references, while the compressive resistances were ~85% lower than the references (Figure 3 and Figure 4). Replacement of 50% of the sand volume with TR grains (sample 6) led to an increase in the mechanical resistances with respect to the composites with 100% sand replacement, due to the presence of the more resistant sand aggregate. In fact, in this case, the flexural strength decrease was approximately 40–55% with respect to both references, while the compressive resistances were ~70% lower than the reference samples [51,53,54,55,56,57]. The specimen with fine tyre rubber aggregates (samples 3) showed higher mechanical resistances than the coarse tyre rubber type (samples 4), as was also observed in previous works [53,54,55,56], a result ascribed to the higher surface area of the fine type elastomeric materials, which, as observed in rheological measurements, improve the cohesiveness of the mixture. Mortars based on PG and PG/TR aggregates (samples 5 and 8) showed an increase in the flexural and compressive strengths with respect to tyre rubber composites, in particular the flexural resistance of sample 5 (PG specimen) was almost double the other tyre rubber samples (samples 3, 4 and 7) together with a three times higher increase of the compressive strength. Moreover, the PG composites showed a decrease in the flexural resistances in the range of 25–50% (sample 5) and 50–60% (sample 8) with respect to the references, and a decrease in the compressive resistances in the range of ~65% (sample 5) and ~80% (sample 8) with respect to the references [58,59,60]. The presence of tyre rubber was detrimental for the mechanical strengths, which conversely were interesting when bare PG was used because glass showed higher stiffness and better adhesion to the cement paste due to the high roughness of the beads and to a chemical composition (silicates, aluminates) similar to the ligand matrix (Figure 5C) [11,61]. For this reason, the lowest specific weight of the glass samples was exclusively ascribed to the intrinsic porosity of the aggregate and not to the porosity of the composite at the interface. Table 3 shows the results obtained from the mechanical tests.

The flexural failure mode of the mortars containing bare TR aggregate did not exhibit the typical brittle behavior observed in the conventional sand-based samples (samples 1 and 2), indeed a separation of the two parts of the specimens was not observed but only discrete cracks, ascribed to the tyre tensile strength (Figure 3, on top) [53,62].

Similar to flexural strength observations, the compressive failure observed in the case of TR mortars was more gradual and the specimens showed a high-energy absorption capacity because of the load retention after failure without collapse. It was also observed that many aggregates of the TR specimens sheared off along the failure plane, and accordingly, the bond between the TR aggregate and the cement paste was stronger than the failure strength of the aggregate granules. Reference samples and sample 5 (based on bare PG) showed a typical brittle failure [60,63,64,65]. A semi-brittle failure was observed in the case of the Sand-TR and of the PG/TR samples (sample 6 and 8). The R_c_ values of the PG, TR_f_/Sand and TR_f_/PG samples were in the CS IV conformity range for plasters, while the R_c_ values of the TR_f_, TR_c_ and TR_f_/TR_c_ samples were in the CS III conformity range for plasters [66]. The R_c_ values of the PG and TR_f_/Sand samples were in the M10 conformity range for masonries, while the R_c_ values of TR_f_/PG, TR_f_, TR_c_ and TR_f_/TR_c_ were in the M5 conformity range for masonries [67].

**Figure 6:**
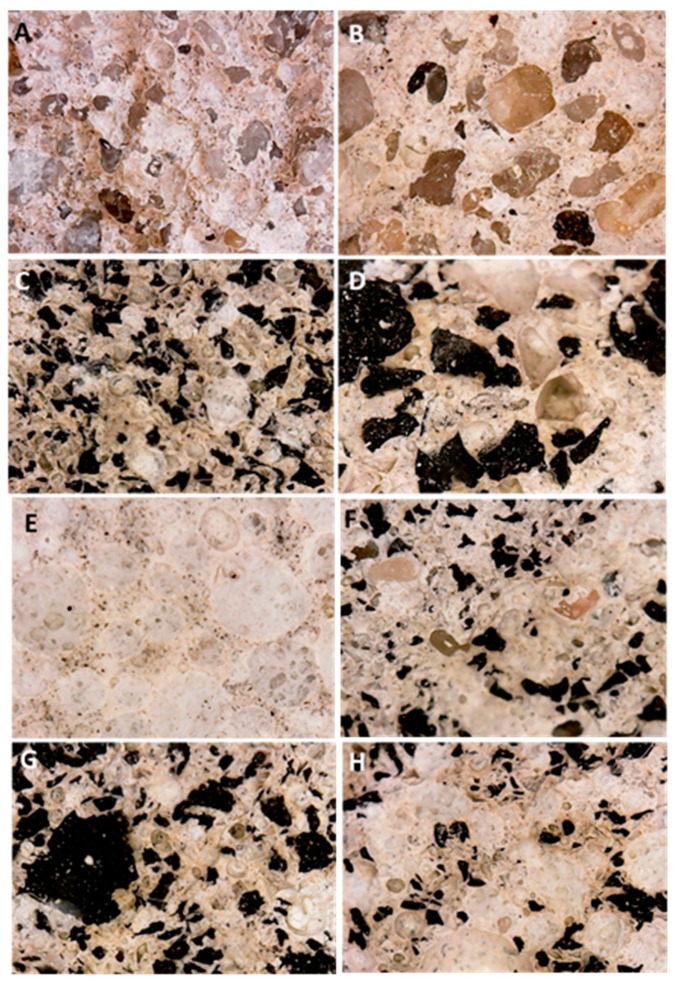
Sections of the specimens after the mechanical tests. (**A**) Norm (sample 1), (**B**) Sand (sample 2), (**C**) TR_f_ (sample 3), (**D**) TR_c_ (sample 4), (**E**) PG (sample 5), (**F**) TR_f_/Sand (sample 6), (**G**) TR_f_/TR_c_ (sample 7), (**H**) TR_f_/PG (sample 8).

Figure 6 shows a picture of the sections of the specimens after the mechanical tests, where the nature of the aggregates can be observed together with a good distribution of the tyre rubber grains, porous glass beads and conventional sand. The samples, although characterized by different types of aggregates and with different grain size distribution, were extremely homogenous without any form of segregation.

**Figure 7:**
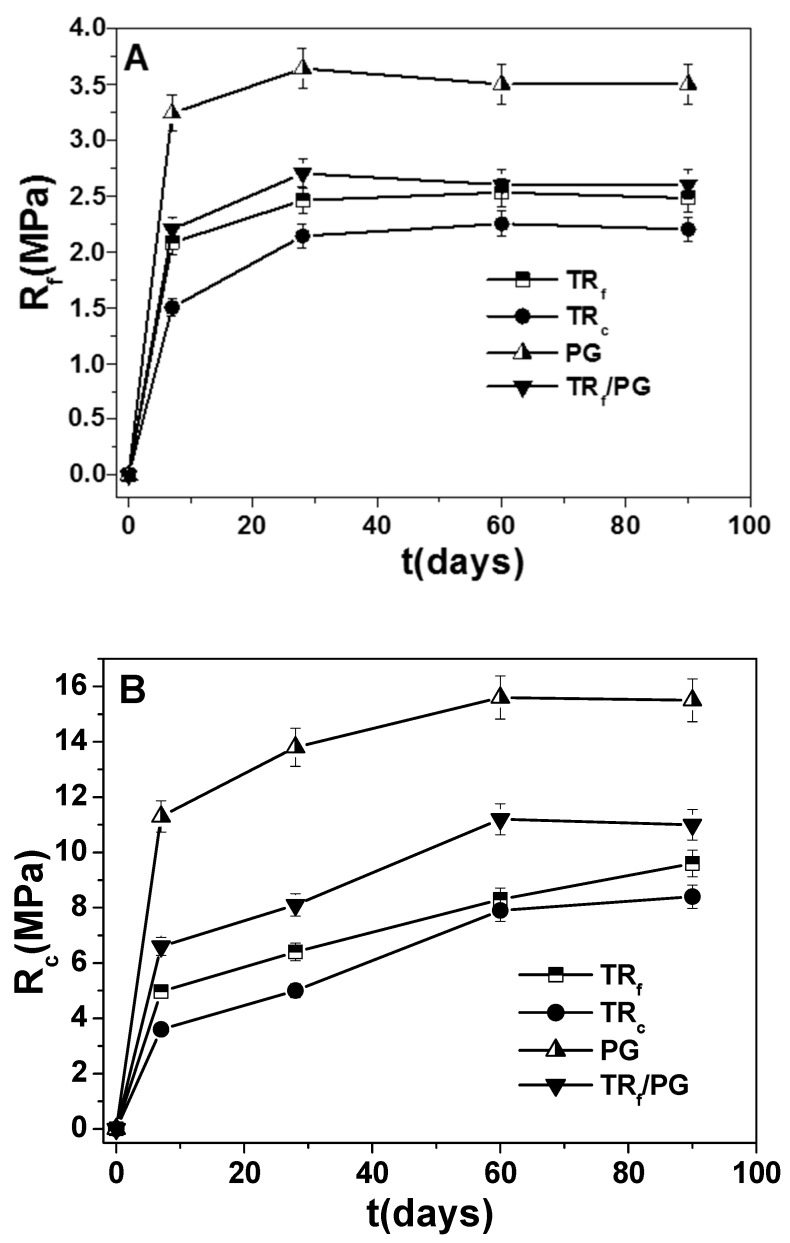
Temporal evolution of the (**A**) flexural and (**B**) compressive resistances of sample 3 (TR_f_), sample 4 (TR_c_), sample 5 (PG) and sample 8 (TR_f_/PG).

The temporal evolution of the flexural and compressive resistances of samples 3, 4, 5 and 8 is reported in Figure 7. An increase in the strengths can be observed for every composite with a final stabilization in the range of 60–90 days. This result may demonstrate a stability of the materials, in consideration of the adopted curing/conservation conditions of the conglomerates (in water). 

**Figure 8:**
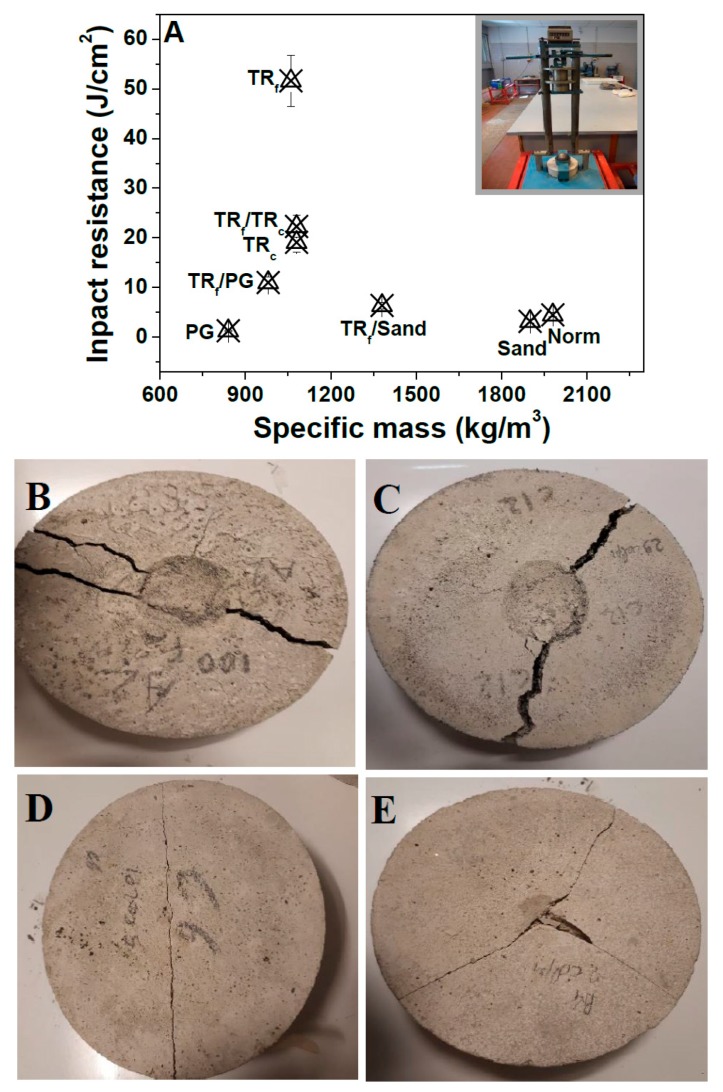
(**A**) Impact resistance of the cement mortars, (**B**) TR_f_ (sample 3), (**C**) TR_f_/TR_c_ (sample 7), (**D**) Norm (sample 1), (**E**) PG (sample 5).

From the impact compression tests (Figure 8A), it was observed that the references and the PG mortars (samples 1, 2 and 5) were extremely fragile, with a relatively low number of blows necessary for fracture formation (Figure 8D,E).

The best results were obtained with the TR specimens because of the load retention of these composites, ascribed to the super-elastic properties of the elastomeric material and evidenced by a deep groove before complete failure (Figure 8B,C) [46,68]. Specifically, sample 3 with finer grains showed the highest energy absorption capacity ascribed to a better compaction with respect to the other similar composites. As for the flexural and compressive tests, average values were observed in samples with 50% of TR (samples 6 and 8) because of the presence of brittle materials as sand and PG. 

### 3.3. Thermal Tests

**Figure 9:**
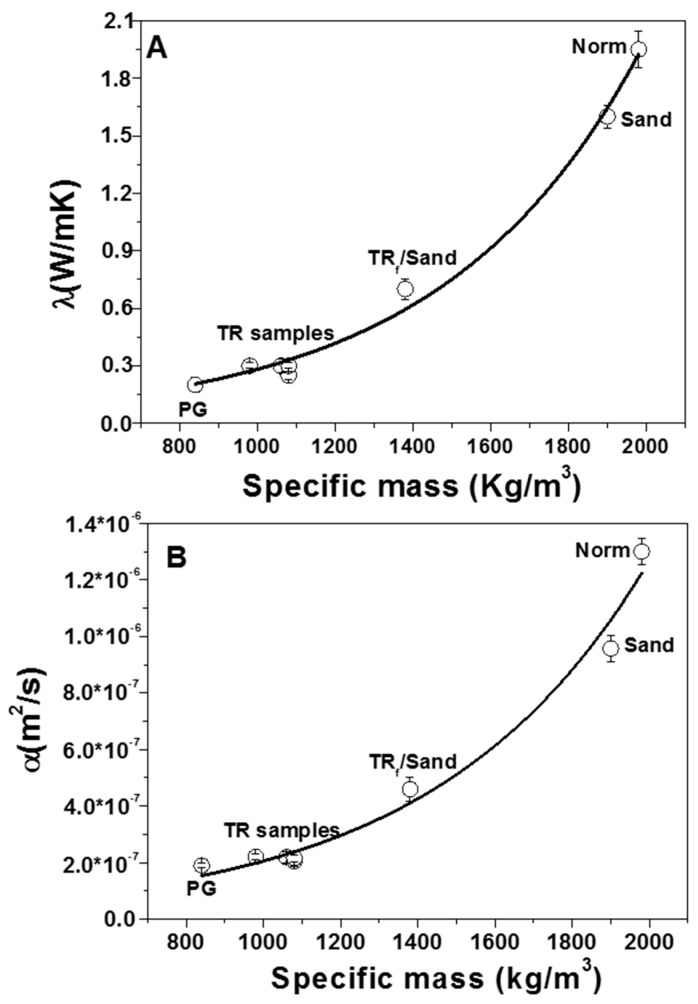
(**A**) Thermal conductivity and (**B**) thermal diffusivity of the cement mortar specimens.

TR-based mortars (samples 3, 4 and 7) showed lower thermal conductivities and diffusivities (80–85%) as compared to the sand equivalent controls (Figure 9) because of the lower specific weight of the specimens due to the polymer characteristics (low specific weight of the aggregate) and also to the voids (entrapped air) at the TR/cement paste interface, which limit heat transport through the material (see Figure 5A) [50,68]. The best results were obtained in the case of the PG mortar (0.2 W/mK, sample 5). Specifically, a corresponding decrease (~90%) of the thermal conductivity with respect to the controls was observed, a result ascribed to the large porosity of the glass beads, which induces a further increase in the thermal insulation (see Figure 1B). In addition, the TR/PG mixture affected the thermal insulation. Average values (60–65%) were obtained in samples with the presence of 50% of sand (sample 6). An exponential decrease in the conductivity and diffusivity data was observed with the decrease in the conglomerates specific weight. Table 3 also shows the results obtained from the thermal tests.

### 3.4. Wettability Tests

**Figure 10:**
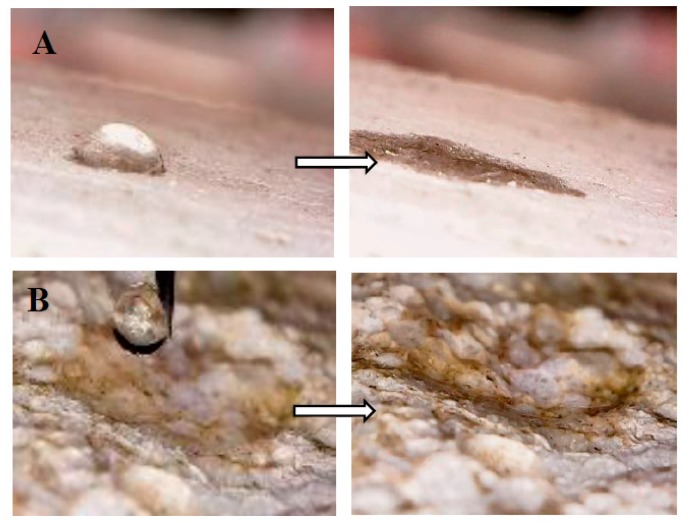

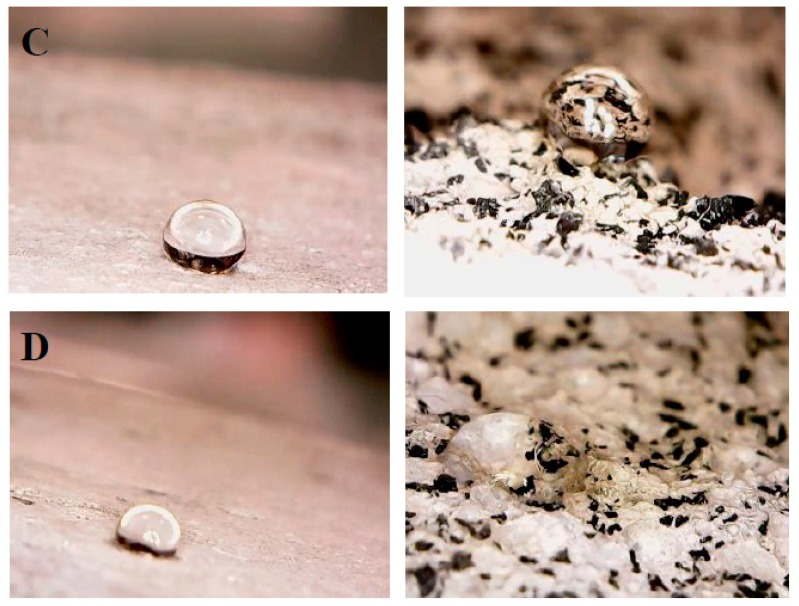
Wettability tests on the side surface of (**A**) Norm (sample 1) at t = 0 s (left) and at t = 60 s (right), on the fracture surface of (**B**) PG (sample 5) at t = 0 s (left) and at t = 2 s (right), on the side (left) and fracture (right) surface of (**C**) TR_f_ (sample 3) at t = 150 s, on the side (left) and fracture (right) surface of (**D**) TR_f_/PG (sample 8) at t = 150 s.

An investigation on the wettability of the tyre rubber specimens was carried out in a previous work [52]. In the present paper, it was completed with the comparison of the properties of the porous glass mortars. As known, the wettability is the ability of a liquid to maintain contact with a solid surface, thus a hydrophobic behavior is associated with surfaces that repel water (poor wettability), while a hydrophilic behavior is associated with a favorable wettability of the surfaces [69]. A surface is considered hydrophobic if the water contact angle is higher than 90°, whereas a surface is considered hydrophilic if the water contact angle is lower than 90°. As has also been formerly observed [52], the surface and the bulk of the sand-based samples (norm and sand) showed an average fast water absorption and a hydrophilic behavior (water contact angle lower than 90°) due to the hydrophilic porous domains of the cement paste (Figure 10A). Similar results were obtained in the case of the porous glass mortar (PG), a result ascribed to the hydrophilic nature and the high porosity of the soda-lime aggregate (Figure 5C), together with the presence of the hydrophilic porous domains of the cement paste, which determine a fast penetration of water (Figure 10B). Tyre-rubber specimens (TR_f_, TR_c_, TR_f_/TR_c_) showed a strong reduction in water penetration both on the surface and on the bulk and a hydrophobic behavior (water contact angle higher than 90° [52]), although these samples were more porous than the references (poor adhesion of the aggregate to the cement paste, see Figure 5A). These results were totally ascribed to the hydrophobic nature of the organic aggregate. Maximum hydrophobic performances were obtained in the presence of the finer tyre rubber grain size distribution (TR_f_, Figure 10C). In addition, in the case of the rubber/sand sample (TR_f_/Sand) the water absorption was significantly lower than the reference samples (~15% on the side surface, ~25% on the fracture surface), but higher than the TR-mortars due to the halved volume of rubber, which dramatically reduced the net force for water penetration and thus stabilized the deposited drops on the surface [52]. Interestingly, the rubber/glass sample (TR_f_/PG) showed a low water absorption (~10% on the side surface, ~15% on the fracture surface) ascribed to the contribution of the organic hydrophobic aggregate in spite of the contribution of the hydrophilic and porous glass (Figure 10D). Therefore, in this case, the rubber contributed to the reduction of the net force for water penetration, thus stabilizing the deposited drops on the surface.

It is worth saying that all materials showed a similar behavior because the surface and bulk of all the specimens were almost the same, which means that the mortar features cannot be modified by eventual wear or damage events of the surface.

## 4. Conclusions

End-of-life tyre rubber (TR) and recycled waste porous glass (PG) were employed for the production of lightweight eco-sustainable cement conglomerates specifically for indoor applications. A cheap and environmentally friendly process was used because the aggregates were not pre-treated. The secondary raw materials were added as partial and/or total replacement of the conventional sand aggregate, which was made on a volume basis rather than on a weight basis due to the low specific weight of both waste materials. The specimens were characterized by rheological, mechanical, thermal, microstructural and wettability tests.

TR and PG were added as partial and/or total replacement of the conventional aggregate, which was made on a volume basis rather than on a weight basis due to the low specific weight of both waste materials. In the present case, the total volume of aggregate was set at 500 cm^3^ in order to preserve an acceptable workability of the mixture. The samples were prepared with a water/cement ratio equal to 0.5, a value that was chosen according to the standard protocol for the normalized mortar preparation, showing a plastic behavior.

The main results showed that: 

(a) Fine TR aggregates affected the cohesiveness of the mixtures as opposite to coarse TR and PG types.

(b) The flexural and compressive strengths of the unconventional and lightweight mortars were lower than the references, based on the more resistant sand aggregate. Specifically, a decrease of ~60% and ~85% respectively of the flexural and compressive strengths was observed in the case of TR samples, whereas a lower decrease was observed in the case of the PG specimens (~25% and ~65% decrease respectively of the flexural and compressive strengths). The flexural and compressive strengths of the PG samples were higher than the TR samples because of the higher stiffness and good adhesion of the glass to the cement paste, ascribed to the roughness of the aggregate surface together with a chemical composition (silicates, aluminates) similar to the ligand matrix. On the contrary, an unfavorable adhesion of the rubber aggregate to the cement paste was observed and ascribed to the hydrophobic nature of the organic material and to the completely different chemical composition of the polymer and of the inorganic matrix.

(c) The specimen with fine tyre rubber aggregates showed higher mechanical resistances than the coarse tyre rubber type, a result ascribed to the higher surface area of the fine type elastomeric materials, which, as observed in rheological measurements, improves the cohesiveness of the mixture.

Replacement of 50% of the sand volume with TR grains led to an increase in the mechanical resistances with respect to the composites with 100% sand replacement, due to the presence of the more resistant sand aggregate.

(d) The flexural failure mode of the mortars containing bare TR aggregate did not exhibit the typical brittle behavior observed in the conventional sand-based samples and in the bare PG samples, indeed a separation of the two parts of the specimens was not observed but only discrete cracks were noticed and ascribed to the rubber tensile strength.

(e) From the impact compression tests it was observed that the references and the PG mortars were extremely fragile, and the best results were obtained with the TR specimens because of the load retention of these composites ascribed to the super-elastic properties of the elastomeric material and evidenced by a deep groove before complete failure.

(f) From thermal measurements it was observed that the thermal conductivity and diffusivity decrease of the lightweight materials (tyre rubber and porous glass specimens) was in the range of ~80–90% with respect to the sand-based samples. This was ascribed to the large porosity of the glass beads and, in the case of the TR specimens, to the voids at the TR/cement paste interface, which limit heat transport through the material. 

(g) Suitable applications in the construction industry as non-structural artifacts may be found for all the samples, as acceptable compressive data for plasters and masonries were obtained. Specifically, after wettability investigations, bare PG specimens may be suitable for indoor applications, while TR specimens may be also suitable for outside elements exposed for example to water flowing and capillary rise. The latter application may also be indicated for the glass/tyre rubber mortars characterized by hydrophobic behavior and low water absorption, ascribed to the presence of the organic aggregate, and by interesting mechanical resistances and high thermo-insulation, mainly ascribed to the inorganic aggregate.

Finally, it is worth considering that these waste/cement composites are cost-effective and environmentally sustainable construction materials because they are prepared through a cheap and eco-friendly process where the aggregates were not pre-treated and the mixture preparation did not require complex manufacturing processes or expensive procedures.

## Figures and Tables

**Table 1:** Mortars composition.

Sample	Cement (g)	Water (g)	Sand Volume (cm^3^)	TR_f_ Volume (cm^3^)	TR_c_ Volume (cm^3^)	PG Volume (cm^3^)
Norm	450	225	810	0	0	0
Sand	450	225	500	0	0	0
TR_f_	450	225	0	500	0	0
TR_c_	450	225	0	0	500	0
PG	450	225	0	0	0	500
TR_f_/Sand	450	225	250	250	0	0
TR_f_/TR_c_	450	225	0	250	250	0
TR_f_/PG	450	225	0	250	0	250

**Table 2:** Type, aggregate composition, specific weight *ρ* and porosity of the cement mortar specimens. Samples prepared with 225 g of water and 450 g of cement. TR_f_ = fine tyre rubber, TR_c_ = coarse tyre rubber, PG = porous glass.

Sample	Type	Aggregate Composition	ρ Kg/m^3^	Porosity %
1	Norm	Normalized sand	1980	22
2	Sand	100% Sand (0.5–2 mm)	1900	25
3	TR_f_	100% TR (0–0.5 mm)	1060	46
4	TR_c_	100% TR (0.5–2 mm)	1080	47
5	PG	100% PG (0.5–2 mm)	840	57
6	TR_f_/Sand	50% TR (0–0.5 mm)/50% Sand (0.5–2 mm)	1380	40
7	TR_f_/TR_c_	50% TR (0–0.5 mm)/50% TR (0.5–2 mm)	1080	45
8	TR_f_/PG	50% TR (0–0.5 mm)/50% PG (0.5–2 mm)	980	52

**Table 3:** Main results from flexural (R_f_), compressive (R_c_), impact resistance (IR) and thermal tests (λ).

sample	R_f (28d)_ (MPa)	R_c (28d)_ (MPa)	IR (J/cm^2^)	λ (W/mK)
Norm	7.2	42.8	4.5	1.95
Sand	5.3	42.6	3.2	1.6
TR_f_	2.5	6.4	51.6	0.3
TR_c_	2.1	5.1	19.1	0.3
PG	3.6	13.8	1.3	0.2
TR_f_/Sand	3.1	10.8	6.4	0.7
TR_f_/TR_c_	2.1	5.5	22.3	0.25
TR_f_/PG	2.8	8.1	11	0.3
